# Ethical Development of Digital Phenotyping Tools for Mental Health Applications: Delphi Study

**DOI:** 10.2196/27343

**Published:** 2021-07-28

**Authors:** Nicole Martinez-Martin, Henry T Greely, Mildred K Cho

**Affiliations:** 1 Center for Biomedical Ethics School of Medicine Stanford University Stanford, CA United States; 2 Stanford Law School Stanford, CA United States

**Keywords:** ethics, neuroethics, digital phenotyping, digital mental health, Delphi study, mental health, machine learning, artificial intelligence, mobile phone

## Abstract

**Background:**

Digital phenotyping (also known as *personal sensing*, *intelligent sensing*, or *body computing*) involves the collection of biometric and personal data *in situ* from digital devices, such as smartphones, wearables, or social media, to measure behavior or other health indicators. The collected data are analyzed to generate moment-by-moment quantification of a person’s mental state and potentially predict future mental states. Digital phenotyping projects incorporate data from multiple sources, such as electronic health records, biometric scans, or genetic testing. As digital phenotyping tools can be used to study and predict behavior, they are of increasing interest for a range of consumer, government, and health care applications. In clinical care, digital phenotyping is expected to improve mental health diagnoses and treatment. At the same time, mental health applications of digital phenotyping present significant areas of ethical concern, particularly in terms of privacy and data protection, consent, bias, and accountability.

**Objective:**

This study aims to develop consensus statements regarding key areas of ethical guidance for mental health applications of digital phenotyping in the United States.

**Methods:**

We used a modified Delphi technique to identify the emerging ethical challenges posed by digital phenotyping for mental health applications and to formulate guidance for addressing these challenges. Experts in digital phenotyping, data science, mental health, law, and ethics participated as panelists in the study. The panel arrived at consensus recommendations through an iterative process involving interviews and surveys. The panelists focused primarily on clinical applications for digital phenotyping for mental health but also included recommendations regarding transparency and data protection to address potential areas of misuse of digital phenotyping data outside of the health care domain.

**Results:**

The findings of this study showed strong agreement related to these ethical issues in the development of mental health applications of digital phenotyping: privacy, transparency, consent, accountability, and fairness. Consensus regarding the recommendation statements was strongest when the guidance was stated broadly enough to accommodate a range of potential applications. The privacy and data protection issues that the Delphi participants found particularly critical to address related to the perceived inadequacies of current regulations and frameworks for protecting sensitive personal information and the potential for sale and analysis of personal data outside of health systems.

**Conclusions:**

The Delphi study found agreement on a number of ethical issues to prioritize in the development of digital phenotyping for mental health applications. The Delphi consensus statements identified general recommendations and principles regarding the ethical application of digital phenotyping to mental health. As digital phenotyping for mental health is implemented in clinical care, there remains a need for empirical research and consultation with relevant stakeholders to further understand and address relevant ethical issues.

## Introduction

### Background

Digital phenotyping tools are expected to improve mental health diagnosis and treatment when integrated into clinical care [1-3]. Digital phenotyping presents significant areas of ethical concern, particularly in terms of privacy and data protection, consent, bias, and accountability [4]. For this study, a modified Delphi approach was used to identify recommendations from panelists with relevant expertise (eg, computer science, mental health care, health law, and ethics) for the ethical application of this emerging technology to mental health.

*Digital phenotyping* refers to new approaches to measure behavior through the collection of biometric and personal data in situ from digital devices, such as smartphones, wearables, or social media. The data are analyzed to generate moment-by-moment quantification of a person’s mental state or prediction of their future behavior [5]. For example, data on pulse rate, finger taps, or voice features can be tracked using an individual’s smartphone and then analyzed to measure behavior, physiological states, and cognitive functioning [6-9]. As the field of digital phenotyping has evolved, projects increasingly include multiple data streams in the analyses, such as data from electronic health records (EHRs), facial recognition technology, ambient sensors, biological scans, or genomic information [10-13]. The proper terminology for these techniques is still under debate, with terms such as *computational behavioral analysis* [14], *continuous measurement* [15], or *personal sensing* also being applied to similar research approaches that involve continuous monitoring of behavioral data gathered from sensors or digital sources [16,17]. Liang et al [18] suggest a broadened definition of digital phenotyping to incorporate the trends toward using multiple data streams, encompassing intelligent systems that sense and mine information related to mental health states “based on the ubiquitous ‘digital footprints’ from multiple data sources, e.g., ubiquitous sensors, social media and healthcare systems.” The term *digital phenotyping* is used in this paper, in part because it was the term used in the Delphi study. Furthermore, the expanded definition of *digital phenotyping* by Liang et al [18] captures the range of ethical concerns regarding the collection and use of data for digital phenotyping projects addressed by the Delphi participants.

Digital phenotyping has a range of health applications, such as the identification of cardiovascular disease risk [19] or suicidal ideation [20]. However, mental health has been a primary area for the investment and development of this technology. Mental health applications of digital phenotyping include analysis of sleep patterns to predict episodes of relapse in schizophrenia [21], early identification of postpartum depression [22], use of keystroke patterns to predict episodes of mania [23], movement or linguistic analysis to predict episodes of depression [24,25], and social media data used to identify drinking and tobacco abstinence behaviors [26]. Mental health applications have been a primary focus of digital phenotyping projects in part because of the ease with which mobile technology can be used to gather massive amounts of fine-grained behavioral data from the user at any time and in any location [27-29]. These types of data are seen as having great potential to address one of the long-standing difficulties in psychiatric research, namely, the lack of definitive biomarkers or objective physiological measures for reliable psychiatric diagnosis [30,31]. Moreover, the collection of psychiatric data had previously been limited to clinical encounters, making it difficult to gather a complete picture of the day-to-day course of behavioral disorders [32]. The advances in technology for collecting and analyzing behavioral data have been applied toward filling this need for better psychiatric research tools.

The consumer domain and institutions such as the military, employers, insurance organizations, and the criminal justice system have also demonstrated a strong interest in the type of behavioral analyses and predictions offered by digital phenotyping [33-35]. The recommendations of the panel focused primarily on clinical applications because it is the domain in which there is primary investment and publications related to behavioral digital phenotyping [36-39]. We also focused on applications in one country (the United States) in order to facilitate the analysis of regulatory implications based on a limited set of regulations and regulatory frameworks. In the United States, clinical applications are *regulated* technologies [40], in that they are subject to government regulation, such as by the US Food and Drug Administration (FDA) or the privacy rule under the Health Information Portability and Accountability Act (HIPAA). Nonetheless, clinical applications of digital phenotyping and associated data collection practices, as explained in detail below, present challenges for the traditional frameworks used for the regulation of data or medical devices. Furthermore, data collection for clinical digital phenotyping may use consumer devices or take place outside of the regulatory frameworks. The Delphi panelists paid attention to ethical concerns relevant to both regulated and unregulated applications for digital phenotyping, because the traditional ethical and regulatory frameworks may inadequately account for issues such as data protection or oversight in digital phenotyping.

This Delphi study was used to address ethical issues raised by mental health applications of digital phenotyping, such as privacy and data protection, consent, transparency, potential for bias in outcomes, and accountability [4]. Digital phenotyping presents novel concerns because the types of data collection and analytics involved are not adequately addressed under current ethical and regulatory frameworks [41,42]. For example, in the health care domain, the FDA is still evolving in its approach to regulating digital software and algorithms [43]. The HIPAA Privacy Rule provides protection for health information collected in health care systems [44]. However, digital phenotyping has the potential to create sensitive health information outside of contexts covered by HIPAA, such as information collected by consumer devices or in settings outside of health care, and the Federal Trade Commission can provide oversight regarding deceptive claims or transparency in relation to consumer uses of digital phenotyping. However, the Federal Trade Commission is limited to the scope of its authority to address broader concerns of safety and privacy in digital phenotyping [45].

Digital phenotyping projects may include many forms of data, from social media, location data, and EHRs to screen taps to genomic data and biometric scans, raising concerns regarding the massive volume of data and appropriately addressing the relevant data protection issues [46]. Under HIPAA, health data that contain personal identifiers can only be shared with third parties when it is used for the purposes of treatment, payment, and health care operations and when a business associate agreement is in place [47,48]. In practice, information in EHRs may be accessible to third parties in ways that patients are not expecting [49]. There have also been examples of third-party companies with whom health care data are shared under business associate agreements and inadequate patient records [50]. Deidentified data (data from which 18 specific identifiers, such as name and age, have been removed) may be shared without restriction under HIPAA [51]. At the same time, owing to advances in computing and the availability of large public databases, reidentification of personal data can be accomplished with increasing ease [52,53]. Thus, there is potential for deidentified patient data that are shared with third parties to later be reidentified and used in ways that the patient could not have foreseen or expected [54].

In the current data landscape, the brokerage of personal data and, more specifically, the sale of behavioral and health inferences that can be generated from those data, is a US $200 billion industry [55]. Outside of the health care domain, privacy protection for personal data varies widely according to jurisdiction and type of data. There has been a gradual movement for more jurisdictions to consider the regulation of personal and biometric data, such as the General Data Protection Regulation in the European Union or the California Consumer Privacy Act [56]. Although these regulations provide a useful model for personal data protection, they are not without shortcomings. For example, existing regulations do not address or sufficiently protect individuals from companies and institutions, drawing health inferences from personal data [57,58]. Furthermore, these data or health inferences may be used in ways that have negative ramifications for people, such as higher insurance rates or employment discrimination [59,60]. Adding further concern, some consumer digital mental health services have also been found to use misleading or false claims regarding their collection and use of sensitive personal information [61]. Against this backdrop, even clinical, *regulated* applications of digital phenotyping present significant concerns regarding transparency, consent, and the distribution of risks and benefits for patients and users regarding how their data may be shared and used.

The algorithms used for many digital phenotyping applications, particularly machine learning algorithms, present additional challenges in terms of the regulation and oversight of these tools. With machine learning algorithms, it can be difficult for those reviewing the machine learning tool to be able to evaluate why the data inputs led to a particular output or findings [62]. This *black box* problem, combined with industry concerns for protection of intellectual property, can make it more difficult to detect and address potential systematic problems in the outputs, such as biases in analyses that disproportionately impact different user populations [63,64]. For that reason, efforts have been made to better define and achieve adequate transparency in health algorithms, as well as calls for *explainability* in algorithms [65]. In terms of regulation, the FDA has been shifting its approach to the regulation of digital medical devices. The FDA’s Digital Software Precertification program is a relatively recent approach in which companies that are certified as having *a robust culture of quality and organizational excellence* are given a streamlined process for product approval [66]. This type of approach has been criticized for needing more clearly defined standards for *excellence*, as well as insufficiently identifying a process for re-evaluation of products that are in use or accountability for maintaining standards [67]. Gerke et al [68] noted that the FDA and European and US regulations of medical devices have been product-based, and thus need to be further adapted to be able to more effectively address the safety and efficacy concerns that machine learning tools present when placed within a health delivery system. In other words, a systems approach is recommended for the appropriate regulation of algorithmic devices in health care settings.

Bias and fairness are concerns for a range of machine learning and digital health technologies [69,70]. Bias can take a number of forms, including a poor fit between the data collected and the research question being asked, data sets that do not adequately represent the target population, and digital tools that may produce disparate effects when applied to different groups [71,72]. Within digital phenotyping specifically, each of the different types of data streams potentially involved, from social media postings to EHR data, may not adequately include people of different racial, socioeconomic, or disability status [73,74]. Furthermore, data used to develop digital phenotyping tools may reflect social inequalities in ways that are difficult to fully account for and address technological fixes. For example, the data in EHRs may reflect physicians’ perceptions and treatment of racialized minorities and associated differential outcomes. There is a need for further research to adequately assess how certain types of digital phenotyping data such as *digital exhaust* may differentially collect information from groups such as people with disabilities or from different racial or cultural groups or different socioeconomic status. Certain predictive uses and applications for digital phenotyping, such as efforts to predict aggression or violence, could be applied in contexts or toward purposes that disproportionately impact marginalized groups. When digital phenotyping tools are not designed or accessible to a range of populations, they can widen gaps in research data or impact mental health diagnosis and treatment in ways that exclude marginalized groups from benefits and even harm those groups [75].

There are a number of efforts underway to address bias in machine learning tools, such as technological fixes to address bias in data sets and algorithms or efforts to provide principles for fairness in algorithmic tools. These are important steps but are unlikely to fully address the many ways in which social inequities may shape the development and results of digital phenotyping tools [76]. For clinical applications, it is important to note that the FDA does not require data regarding the diversity in training data for machine learning tools. A recent review of machine learning health care devices approved by the FDA found that of 130 tools, most did not report whether they had been evaluated at more than one site, and only 17 included demographic subgroup evaluations in their submissions [77]. The digital divide in digital phenotyping devices could further exacerbate inequities in the distribution of risks and benefits in mental health care.

In clinical and health research settings, consent procedures will need to adequately inform individuals of when and how their data are being gathered and used, as well as whether and how they may receive notice of the findings or repercussions of the digital phenotyping analyses. For digital phenotyping, consent challenges include the difficulty of adequately explaining the probabilistic nature of findings, as well as the potential ramifications from personal data or the inferences that may be drawn from seemingly mundane data such as screen taps or location. The complexity of digital phenotyping findings, as well as the potential ramifications from the data and health inferences generated, can be difficult to convey. Although these consent issues overlap with those applicable to genomic research, some differences are the shorter timeframe for digital phenotyping predictions (eg, risk of a psychotic episode in the next month), more direct responsibility placed on patients to modify their behavior immediately, and the potential for a person’s results to be shared or used in domains outside of health care. In addition, there are considerations of appropriate transparency and informed consent for the use of digital phenotyping tools in vulnerable populations, such as children and older adults [78]. As an early intervention in psychiatric conditions generally improves treatment outcomes, mental health research often aims to identify indicators of severe mental illness in early childhood and adolescence [79,80]. Informed consent and transparency procedures for digital phenotyping in children will need to be sensitive to the potential negative impacts of returning predictive results to young people and take into account children’s rights to autonomy and parental interest in being informed [81].

The clinical use of digital phenotyping tools is also thought to have the potential to disrupt the traditional patient-therapist relationship. Artificial intelligence tools are thought to have the potential to disrupt or even replace some of the roles traditionally held by therapists or clinicians [82-84]. The use of artificial intelligence methods, such as machine learning and natural language processing, is thought to raise issues of whether the device’s findings will be viewed by physicians and patients as more objective than as physician judgment or patient’s self-report, thus intruding upon the therapeutic relationship. In instances where a device’s recommendations differ from the physician’s judgment, there are concerns regarding liability and accountability for any errors in the tool’s findings, as well as the nature of the fiduciary relationships involved [85].

### Objectives

The Delphi technique is a widely used method for engaging a group of experts to identify and explore a range of approaches to a policy issue, potentially establish areas of convergence and consensus among the recommendations and reveal key assumptions or correlations for different judgments [86,87]. The purpose of this modified Delphi study is to identify priority issues of ethical concern in the development of mental health applications using digital phenotyping and areas of agreement regarding principles for approaching the ethics of digital phenotyping.

## Methods

### Overview

**Figure 1:**
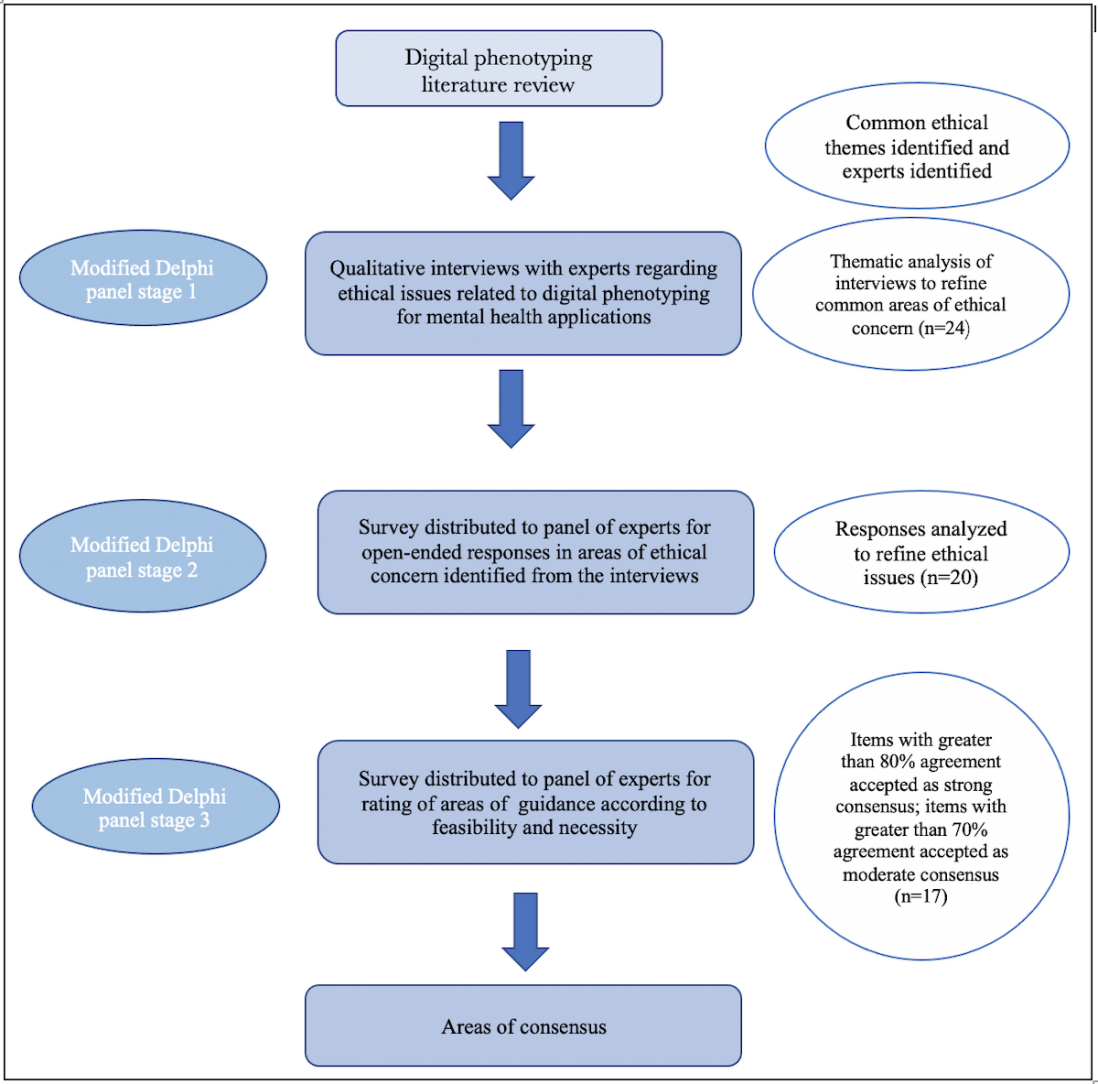
Delphi study overview.

The Delphi technique is essentially a method of structuring communication among a group of people with relevant expertise to discuss resolutions to a complex problem [88,89]. Although many modifications to the Delphi technique have evolved over time, the main features of this method include (1) anonymity of the panelists, meaning they do not know of each other’s identities or which panelist provided which answers, in order to avoid the influence of status or personality on the discussion; (2) controlled feedback, in which the panelists’ answers are given to the study coordinator who then processes and disseminates the resulting information; and (3) an iterative process in which experts are consulted more than once to give them the opportunity to reconsider and refine their views [90]. For this study, the modified Delphi technique was used in the stages depicted in Figure 1. This study was designated as exempt by the local institutional review board.

We recruited experts to represent areas of stakeholder relevance in digital phenotyping: (1) computer science, (2) psychiatry and mental health therapy, (3) law, (4) ethics, and (5) lived mental health experience. The category of people with *lived experience* refers to people who have a diagnosis of mental illness. Inclusion of this area of expertise was meant to provide a fuller perspective on the potential ethical impacts of digital phenotyping [91]. For this category, we also looked for people with some experience in mental health advocacy or policy as a foundation for discussing potential ethical issues, such as privacy or consent, relevant to digital phenotyping.

### Composition of the Delphi Panel

We searched the PubMed, Google Scholar, and LexisNexis databases to identify people in industry and academia developing technology in the area of digital phenotyping. Search terms included *digital phenotyping*, *personal sensing*, *computational behavioral analysis*, and *behavioral analytics*. The literature review also yielded specific subareas of expertise relevant to computer science, ethics, and law, relating to emerging technologies, privacy, data protection, machine learning, and bias. Within areas of expertise 1-4, there are also these subareas of expertise represented. For example, in computer science, we included people who worked directly with digital phenotyping as well as those who had related expertise in machine learning, data science, or predictive analytics; within law, we identified people with subspecialties in health, data, and health technology law.

There is no established optimal number of experts for a Delphi panel [92]. Primary factors in deciding on the size of a Delphi panel are appropriate representations of variations in judgment among those with expertise and the drawbacks involved in managing multiple surveys, such as decreasing response rates and increased time needed by researchers in between rounds [93]. Most Delphi studies have used between 15 and 20 participants [94]. In this study, 28 people with relevant expertise were identified through the review process and invited to participate in the qualitative interviews for the first stage of the Delphi study. There were fewer people with lived experience represented on the panel than in the other categories. This reflects a smaller pool of potential panelists that we were able to identify through our search than for other categories. We identified 8 people who were in contact with their participation; 3 people responded to our invitations, and one of those 3 subsequently decided not to proceed with scheduling an interview for personal reasons unrelated to the study itself.

### Interview and Survey Stages

The qualitative interviews were semistructured and explored questions regarding participants’ views of the ethical issues presented by digital phenotyping for mental health applications. Interview transcripts were reviewed to identify the main ethical themes found in expert panelists’ interviews as well as the main areas of their recommendations to address these ethical concerns [95]. The transcripts of the interviews were generated as Microsoft Word documents. An *identifying-category* strategy for reviewing the transcripts and determining the preliminary codes was used [96]. The content of the interviews involved the participants directly referencing ethical categories relevant to health technologies, such as consent or privacy, which facilitated the identification of relevant categories from the transcripts. Once the preliminary categories for the transcript themes were established, we reviewed the transcripts to confirm the categories and identify associated recommendations for addressing the areas of ethical concern. We then used the main ethical themes to generate an open-ended qualitative survey that we distributed among the panelists in which we asked them whether they thought that the identified ethical issue was relevant to digital phenotyping for mental health applications and whether the recommendations to address that issue were appropriate.

Narrative comments from the qualitative survey were used to assist in drafting the statements for the second survey relating to recommendations for ethical mental health applications of digital phenotyping. The second survey was conducted with the same panel of experts who responded to the first survey. For the second survey, we asked panelists to rate statements according to the necessity of a particular recommendation or guidance statement on a four-point scale (1=strong agreement, 2=moderate agreement, 3=neutral, and 4=disagreement). In the second survey, we used a cut-off of 80% rating agreement to indicate strong agreement among panelists, and we deemed 70% moderate agreement with respect to consensus, consistent with the methodology in the Delphi literature [85,97].

Panelists were also asked to rate statements according to feasibility on the same four-point scale. *Feasibility* refers to the likelihood that a particular recommendation could be effectively implemented. During the interview stage, some participants noted that there were some potential recommendations for addressing ethical issues in digital phenotyping that were infeasible. For example, a recommendation for data protection regulation might be identified as desirable but unlikely to be implemented. In some cases, a panelist’s specific expertise provided them with additional insight into the feasibility of an option that is different. In Delphi studies applicable to health care, including ratings for both necessity and feasibility, were found to be more useful for identifying recommendations that could be effectively implemented [98]. For these reasons, we assessed both the necessity and feasibility.

## Results

Of the 28 invitations, 24 (86%) participated in the qualitative interviews, 20 (71%) participated in the first survey, and 17 (61%) participated in the second survey (Table 1).

**Table 1:** Expertise represented at each stage^a^.

Stage	Computer science, n (%)	Psychiatry or therapy, n (%)	Law or ethics, n (%)	Lived experience, n (%)
Interviews (n=24)	8 (33)	8 (33)	9 (38)	2 (8)
Survey 1 (n=20)	6 (30)	6 (30)	7 (35)	2 (10)
Survey 2 (n=17)	5 (29)	6 (35)	7 (41)	2 (12)

^a^Some panelists had expertise in more than one area.

The main ethical concerns that emerged from the qualitative interviews were (1) privacy and data protection, (2) transparency, (3) consent, (4) reporting of findings or return of results, (5) oversight and accountability, (6) fairness and bias, and (7) validation of digital phenotyping tools. Although panelists identified return of results as a potential area of concern, the specific issues identified overlapped heavily with the types of concerns and recommendations aimed at the consent process for digital phenotyping, such as the need to inform patients of the types of results to expect. The panelists also generally did not go further in providing specific recommendations for the return of results beyond what needed to be discussed in consent, as those particulars were seen to be more dependent on the context of the digital phenotyping application.

In the first survey, the panelists were presented with the ethical categories and then asked to provide additional feedback concerning priority areas of ethical concern within those categories and additional details for recommendations to address those concerns. Those areas of concern and associated recommendations were then presented as statements in the second survey for the panelists to rate. Table 2 presents the results of the Delphi method. The statements in the table present the ethical issues in digital phenotyping for mental health applications resulting from the interviews and first survey. The agreement rating listed in the table represents the level of consensus for statements that were determined through the second survey.

**Table 2:** Consensus statements on ethics of mental health applications of digital phenotyping.

Statement^a^			Agreement level^b^		
			Necessity		Feasibility
**Evidence of validity for the intended use**					
	Algorithms incorporated into a digital phenotyping tool, especially at a large scale, have to be thoroughly evaluated in terms of performance and accuracy, including false positives and false negatives.	Strong		Strong	
	Implement processes for review of digital phenotyping tools’ effectiveness after implementation, including review of updates, and monitoring and reporting of adverse events caused by an algorithm’s findings.	Strong		Moderate	
	Digital phenotyping tools that are intended for use in health care should use relevant standards for data systems to support the goal of interoperability with existing health data systems.	Strong		Moderate	
	Digital phenotyping tools for mental health applications should respond to real-world needs and concerns of the intended users, such as clinicians, patients or consumers, in order to enhance user engagement and provide value.	Strong		Strong	
**Transparency**					
	Explanations of the processes, risks, limitations, and results that are relevant to different stakeholders should be provided to them in an appropriate format and reading level.	Strong		Strong	
	Processes involved in the collection, storage, and dissemination of raw data, as well as data processing and the architecture of the algorithms, should be explainable.	Strong		Moderate	
**Accountability**					
	Development and use of digital phenotyping tools (eg, plans for data collection or validation) should be reviewed for potential ethical issues by an independent interdisciplinary group with relevant expertise, starting early in the development process.	Strong		Moderate	
	Provision of appropriate educational and training materials for IRBs^c^ handling review of digital phenotyping projects is also necessary.	Strong		Moderate	
**Consent**					
	Consent should be required from individuals when their personal data are collected for digital phenotyping tools.	Strong		Moderate	
	Consent for collection of digital phenotyping data should include information at a sixth-grade level regarding the types of data collected, the inferences that can be drawn from the data, the reports made from the data, who the data and reports would be shared with, the potential risks and benefits to the user, and the limitations that apply to the findings.	Strong		Moderate	
	Include relevant stakeholders in efforts to formulate and disseminate relevant information for disclosure (eg, data storage, utilizing appropriate languages and formats for relevant stakeholders, such as health care providers, government institutions, advocacy organizations, patients, consumers, or the public).	Strong		Strong	
**Data security and privacy**					
	Data and findings that are identifying should not be collected, used or shared with third parties without the informed consent of that individual.	Strong		Strong	
	Sharing of data to advance scientific research and the validity of the tools remains an important goal.	Strong		Moderate	
	If data will be shared with third-party researchers, clear information, written at sixth-grade reading level, must be given to the individual user about third-party researcher and how they plan to store, use and/or share the data.	Strong		Strong	
	The individual user also must have an option to opt out of sharing their data with third parties.	Strong		Moderate	
	Raw data that is nonidentifying, and nonidentifying summary statistics, may be shared without consent.	Strong		Strong	
	There should be periodic review to re-evaluate whether identifying information can be drawn from the raw data, particularly when combined with other available data.	Moderate		Moderate	
	Raw data should always be encrypted when stored or transmitted; potential identifiers in data (eg, phone numbers and IP addresses) should be replaced with surrogates (eg, hashed or encrypted).	Moderate		Moderate	
	Standards and approaches to minimize risk of reidentification of individuals, such as differential privacy measures, should be implemented.	Strong		Moderate	
	The security standards for data storage, sharing, and use of the individual’s data, as well as the process for monitoring compliance with these standards, should be clearly defined and communicated to users of digital phenotyping tools.	Strong		Moderate	
	Security reviews and audits of data practices should also be implemented.	Strong		Moderate	
**Fairness**					
	Encourage collaborative research and partnerships to develop ways to identify and minimize bias or discrimination in the development of digital phenotyping tools and to identify and minimize any potential bias that may occur because of how the tools may be used in different communities or local contexts.	Moderate		Moderate	
	Conduct research into and implement methods to mitigate bias in different levels of algorithm development, including in the training data, in the algorithmic process or focus, in the transfer of digital phenotyping tools to different contexts, and in the interpretation of digital phenotyping findings.	Strong		Strong	
	Identify the specific ways that mental health and clinical care may impact the potential for bias in these areas. Periodic review and re-evaluation of the methods for addressing and mitigating bias at the different levels of algorithmic development may be needed.	Strong		Strong	

^a^The statements represent the ethical issues in digital phenotyping for mental health applications resulting from the interviews and the first survey.

^b^The agreement rating listed represents the level of consensus for statements that were determined through the second survey.

^c^IRB: institutional review board.

## Discussion

### Principal Findings

The results of this study showed strong agreement for several ethical issues in the development of digital phenotyping: privacy, transparency, consent, accountability, and fairness. Agreement was strongest when the guidance statements were broad enough to accommodate a range of applications. The panelist comments for the survey indicate that the consensus around broader principles reflects the need to allow flexibility for specific contexts and projects for which digital phenotyping might be used for mental health purposes.

The privacy and data protection issues that Delphi participants found particularly concerning generally related to the perceived inadequacies of current regulations and frameworks for protecting sensitive personal information and the potential for sale and analysis of personal data outside of health systems. Most of the participants noted in the interviews that additional data regulation would most likely be necessary to fully address the privacy concerns posed by digital phenotyping. However, advocating for specific technological standards or regulatory measures was seen as beyond the scope of what the panel could meaningfully address. The panelists focused on addressing general principles for privacy and data protection rather than on specific technological standards or regulatory measures.

Clinical digital phenotyping applications are subject to the security and privacy provisions of HIPAA. Nonetheless, panelists noted that digital phenotyping tools may involve data or be applied outside of contexts for which HIPAA or other personal data protections currently apply. As one panelist stated, “HIPAA criteria don’t include new forms of identifiable data like keystroke kinematics - principles and practices need to be more sophisticated to address digital health tech.”

Digital phenotyping poses specific concerns regarding privacy because much of the raw data that are collected, such as screen taps or location data, may not be information that patients or users consider sensitive personal information. Thus, patients and users may not be aware of or be able to foresee how that data may be analyzed to reveal information about their mental state that they would want to keep private.

Transparency and consent were seen as key areas for presenting patients and users with information about privacy and data protection. For the clinical use of digital phenotyping, informed consent would need to include careful consideration of how to communicate the risks and benefits and what, how, and when findings would need to be reported afterward. At the same time, as 2 of the panelists noted in the first round of surveys, providing information effectively can be difficult, especially as patients and users feel that there is too much consent information being given to them and feel overwhelmed or prefer to ignore it. Owing to the complexity involved in collecting data, generating results, and understanding downstream health and data implications, the achievability of complete informed consent is arguable. All panelists agreed that stakeholders should be included in collaborative processes to determine what information should be included in the consent and return of digital phenotyping results.

The study found strong agreement regarding the need for consent for the collection and use of raw data. Increasingly, owing to advances in data science and the availability of massive public databases, personal data can be reidentified [99]. Furthermore, health inferences can be generated from seemingly mundane personal information that can have repercussions for individuals and groups [100]. Existing frameworks under HIPAA distinguish personal health information from deidentified data, with no consent required for the use of deidentified data [101]. However, one panelist stated, “Raw data is not able to be ‘non-identifying’ and consent should be a norm when using or sharing personal data that has potential health implications.”

Another panelist noted, “consent is not needed for analysis of deidentified data by a trusted entity; but public information about the process, including return of aggregate results, is essential.”

One panelist stated in feedback, “All information that is deidentified should not be ‘fair game’ for any uses and disclosures without consent. This is a flaw in the Common Rule. Sensitive and stigmatizing information may be attributable to socially vulnerable groups.”

A different panelist noted that “with respect to the use of personal data in digital phenotyping, it will likely require extensive education of the public to increase data and technology literacy. Developing public trust should be a priority and engaging the public as partners in this endeavor is critical and, expensive.”

Most panelists noted that digital phenotyping for mental health presented significant privacy challenges outside the clinical domain, especially in terms of consumer applications. Given the lack of sufficient relevant privacy regulation or consent requirements in the consumer domain, the panelists did not address potential consumer consent requirements. However, recommendations for transparency regarding the design and data practices for digital phenotyping projects were viewed as a way to address privacy concerns. The panelists agreed that information regarding the collection, storage, and dissemination of raw data should be available to users. Reports regarding the findings of digital phenotyping tools should also be available to users. Such information would need to be available at an appropriate reading level, such as a sixth-grade reading level for users.

As many institutional review boards may not have members with expertise in data privacy or predictive algorithms, the need for institutional review boards to have access to adequate educational materials was noted. Panelists also agreed that independent ethics review of digital phenotyping was useful but what that means in practice could take different forms, with emphasis being on the need for such reviews to have transparent processes and independence in their judgment.

Although clinical applications of digital phenotyping are subject to FDA oversight of validation and safety, the panel identified some specific concerns regarding validating tools for specific contexts and applications. Standards for evaluating validity, accuracy, and effectiveness for specific uses, as well as the mechanisms for performing these evaluations, are still evolving and vary across different contexts. There was consensus regarding the need to have a mechanism for review or auditing of the validity of digital phenotyping tools beyond their initial deployment, such as evaluating software updates or device uses deployed in new contexts. There was also general agreement regarding the need for a mechanism through which the data processing and architecture of the digital phenotyping algorithms could be available for independent third-party reviews. However, a statement that set out a proposal for a *continual review* of digital phenotyping devices received feedback from several panelists, indicating that it would be too burdensome to have such a requirement. Decisions concerning how often and in what situations to re-evaluate a device would depend on usage contexts and specific projects. Survey feedback also noted that *evaluation* of an algorithm used for digital phenotyping could entail different degrees of thoroughness. Thus, these types of specific details regarding evaluation were seen as the domain of professional organizations to establish appropriate technical standards for the evaluation of specific types of devices. Another panelist stated that *explainability* for the algorithms could be a desirable goal, but it is not something that would be feasible to require currently.

The issue of interoperability posed another area in which panelists agreed upon necessity but not upon feasibility. *Interoperability* refers to the ability of data systems and services to have clear, shared standards for the content, context, and meaning of data [102]. Most panelists viewed the ability of data to be used by different systems as necessary to facilitate scientific research using digital phenotyping data. At the same time, comments made in the survey by panelists with computer science expertise noted that although interoperability is a desirable goal, it has encountered practical challenges for implementation in health data that would make it impracticable to put forth as a requirement [103].

The lack of diversity in research participants and the data used for research, such as lack of panelists according to race, gender, or disability, presents concerns for ensuring equity and fairness in digital phenotyping for mental health. The potential for bias needs to be addressed at the different stages of the development process for digital phenotyping tools, from how the initial research questions are formulated, how data are selected and used within these stages, and the potential for disparities resulting from implementation of these tools in different contexts. In particular, practices during the design and development processes are needed to ensure that digital phenotyping tools can be used in different communities and contexts while mitigating potential harm to populations, such as marginalized racial, linguistic, or socioeconomic groups. There was strong agreement regarding the need to address bias and fairness; however, as one panelist stated, “To assess feasibility, context is important and - depending on context, there will be unique barriers and facilitators to implementation.” Another panelist noted that it is “[h]ard to predict where bias might arise, thus this is challenging work, requiring constant vigilance.” Organizations such as the American Medical Informatics Association have been working on specific standards and principles for addressing bias and fairness in algorithms [104-106]. The Delphi panel identified some practices in the development of digital phenotyping that can be useful in identifying areas of potential bias, such as having diverse research teams and engagement of key stakeholders at different stages of the development process.

### Limitations

Although this study met the stated recommendations regarding the size of the Delphi panel and selection of experts, given the size of the panel, some relevant viewpoints might not have been included in the panel. As noted in the *Methods* section, despite efforts to recruit additional panelists who have lived mental health experience, we had a notably smaller number of panelists in that category and thus did not have the benefit of additional insights from that perspective. 

The digital phenotyping literature review raised several areas of ethical concern that were not directly engaged by the Delphi panel in the consensus statements. For example, concerns regarding the potential impact of digital phenotyping on the therapeutic alliance or the impact of continuous monitoring on the experience of patients and participants were not addressed in the consensus statements. This Delphi approach was not intended to comprehensively address all of the potential ethical concerns regarding mental health applications of digital phenotyping. The Delphi process served to identify priority areas of ethical concern for an emerging technology. The consensus aspects of the approach meant that there were relevant ethical issues that did not ultimately be prioritized for inclusion in the recommendations. Nonetheless, excluded ethical concerns, such as impact on the therapeutic relationship, remain relevant and merit scrutiny and empirical research as mental health applications of digital phenotyping become more common.

### Conclusions

This Delphi study found agreement on a number of ethical issues to prioritize in the development of digital phenotyping for mental health applications. Standards and guidelines for key areas of digital phenotyping, such as privacy and data protection outside of health care institutions and the regulation of digital medical devices, are still evolving. The Delphi consensus statements identified general recommendations and principles regarding the ethical application of digital phenotyping to mental health. As digital phenotyping for mental health is implemented in clinical care, there remains a need for empirical research and consultation with relevant stakeholders to further understand and address relevant ethical issues.

## Abbreviations

EHR: electronic health record
FDA: Food and Drug Administration
HIPAA: Health Information Portability and Accountability Act
